# Mechanical properties and sustainable bacterial resistance effect of strontium-modified phosphate-based glass microfiller in dental composite resins

**DOI:** 10.1038/s41598-023-44490-z

**Published:** 2023-10-18

**Authors:** Hye-Bin Go, Myung-Jin Lee, Ji-Young Seo, Sung-Yun Byun, Jae-Sung Kwon

**Affiliations:** 1 Department and Research Institute of Dental Biomaterials and Bioengineering, Yonsei University College of Dentistry, 50-1 Yonsei-Ro, Seodaemun-Gu, Seoul, 03722 Republic of Korea; 2 Department of Dental Hygiene, Division of Health Science, Baekseok University, Cheonan, Republic of Korea; 3 Department of Orthodontics, Institute of Craniofacial Deformity, Yonsei University College of Dentistry, Seoul, Republic of Korea; 4 BK21 FOUR Project, Yonsei University College of Dentistry, Seoul, Republic of Korea

**Keywords:** Biomaterials, Preclinical research, Dentistry, Dental materials, Oral microbiology, Preventive dentistry

## Abstract

Dental composite resins are widely used in dental restorations. However, their clinical application is limited by the occurrence of secondary caries. Strontium-modified phosphate-based glass (Sr-PBG) is a material known to have a sustainable bacterial resistance effect. The mechanical properties (in particular, flexural strength, modulus of elasticity, and hardness) of dental materials determine their function. Therefore, this study aimed to investigate the mechanical and ion-releasing properties as well as the sustainable bacterial resistance effect of bioactive resin composites containing Sr-PBG. The data were analyzed by ANOVA and Tuckey’s tests (*p* < 0.05). We incorporated a Sr-PBG microfiller at 3, 6, and 9 wt.% concentrations into a commercially available composite resin and investigated the mechanical properties (flexural strength, elastic modulus, and micro hardness), ion release characteristics, and color of the resultant resins. In addition, we examined the antibacterial effects of the composite resins against *Streptococcus mutans* (*S. mutans*). The mechanical properties of the Sr-PBG groups differed only slightly from those of the control group (*p* > 0.05). However, the optical density at 600 nm of *S. mutans* incubated on the experimental group was significantly lower compared to that observed with the control (*p* < 0.05) both before and after thermocycling between 5 and 55 ℃ for 850 cycles (dwell time: 45 s). Therefore, strontium-modified resin materials exhibited a sustainable bacterial resistance effect in vitro while maintaining some of the mechanical properties of ordinary acrylic resins.

## Introduction

In recent years, resin-based composites have become the preferred choice of restorative materials because of their low toxicity, high biocompatibility, superior aesthetics, and ease of handling compared to amalgams^1–3^. Over the last 30 years, resin restorative materials have been widely used in both anterior and posterior oral restorations. Nevertheless, as with direct restorations, secondary caries and discoloration adjacent to restorations remain the main reasons for the replacement of tooth restorations^4, 5^. As secondary caries lead to the failure of resin restorations, various strategies have been explored to develop new materials with excellent antibacterial properties since the introduction of resin-based composites in dentistry^6^.

To date, many studies have evaluated composite resins added with antibiotics, fluorine, silver ions, and zinc oxide ions for imparting antibacterial properties^7–10^. However, bacteria-resistant additives tend to affect the structure of resin-based dental materials. The structural changes influence the stability and overall performance. The dynamic oral environment is complex with hydrothermal and mechanical insults, which compromise the therapeutic effect of restorative composites^11, 12^. Hence, the modifications of the composite resin structure should account for structural compatibility while improving the functional effect (e.g., bacterial resistance). Phosphate-based glass (PBG) is a glass network-forming agent based on P_2_O_5_ that contains CaO and Na_2_O. PBG is suitable for use in biomaterials and tissue engineering because the calcium and phosphorus contents of bioactive glasses play an important role in the remineralization process^16^. It is known that the main components of most phosphate-based glass are also found in the bone's inorganic stage^17^. Therefore, PBG has a long-term effect because its chemical composition is close to that of natural bones and teeth, and it slowly decomposes over a period of 1–2 years^17–19^. In particular, phosphate is known to significantly contribute to the formation of hydroxyapatite (HA) and increase biocompatibility^20, 21^. Strontium is one of the various ion species released from conventional and resin-modified glass ionomer cements, and it is known to be associated with reduced acid production by *S. mutans* in biological membranes^22, 23^.

Previous studies on developing antibacterial dental restorative materials, such as flowable composite resins, have focused on the effect of adding strontium ions or bioactive glass to them^24–27^. However, only a few studies have investigated the sustainable bacterial resistance effects of incorporating Sr-modified PBG (Sr-PBG) into composite resins. Therefore, we hypothesized that the addition of Sr-PBG can impart effective bacterial resistance. We investigated the flexural behavior, surface micro hardness, ion-releasing profile, and bacterial resistance effect to test the null hypothesis that a progressive addition of Sr-PBG will improve bacterial resistance while preserving the bioactive effect. Additionally, we also validated the hypothesis by testing the Sr-PBG composite resin before and after hydro-thermal fatigue.

## Materials and methods

### Phosphate glass preparation

To prepare the glass powder, high-purity oxides of P_2_O_5_ (50 mol%), CaO (15 mol%), Na_2_O (20 mol%), and SrO (15 mol%) used as precursors were mixed to homogeneity in a tubular shaker mixer for 60 min. Subsequently, the mixed batches were melted in an alumina crucible at 1100 °C for 1 h, and the melted glass was quenched at room temperature to obtain a glass cullet. The obtained glass cullet was ground in an alumina mortar and then pulverized under dry conditions using a planetary mono mill (Pulverisette-7, Fritsch, Idar-Oberstein, Germany).

The particle size of the so-obtained Sr-PBG powder was analyzed using a particle size analyzer (Mastersizer 2000, Malvern Instruments, UK) and the morphology was observed using a field-emission scanning electron microscope (FE-SEM; JSM-7800 F, JEOL, Akishima, Tokyo, Japan) with an energy-dispersive X-ray (EDX) spectroscope. The amorphous structure of Sr-PBG was confirmed by X-ray diffraction (XRD) analysis (Rigaku, Tokyo, Japan).

### Preparation of Sr-PBG-incorporated composite resin

In this study, a commercially available composite resin (Filtek Z350XT, 3 M ESPE, St. Paul, Minnesota, USA) was used. The experimental groups were prepared by homogeneously mixing the Sr-PBG powder in various proportions (3, 6, and 9 wt.%) with the composite resin, and the original composite resin without Sr-PBG was used as the control group (0 wt.%). The compositions of the control and experimental groups are listed in Table 1, along with the sample codes. All specimens were prepared prior to polymerization, and the Sr-PBG powder was mixed with the composite resin using a speed mixer (DAC 150.1 FVZ, Hauschild Speed Mixer, Hamm, Germany). After being mixed, each sample was polymerized using a light-emitting diode (LED) light-curing device (Elipar S10; 3 M ESPE, Seefeld, Germany). The number of samples was determined according to previous studies^4, 24^. The sample size was calculated according to the Cochrane formula and ISO standard^4^.

**Table 1:** Compositions of the composite resins (control and experimental groups) used in this study.

Group	Group code	Resin (wt.%)	Sr-PBG (wt.%)
1	Control	100	0
2	3 wt.% Sr-PBG	97	3
3	6 wt.% Sr-PBG	94	6
4	9 wt.% Sr-PBG	91	9

### Flexural strength and modulus

The flexural strength and modulus of the composite resins were measured according to ISO 4049. Seven rectangular specimens of size 25 mm × 2 mm × 2 dimensions (length × width × height) for each group were prepared without air bubbles or voids. All samples were polymerized using the LED light-curing unit (Elipar S10, 3 M ESPE, Seefeld, Germany) at five irradiation regions (20 s each) that slightly overlapped on both sides and then stored in distilled water at 37 ± 1 ℃ for 24 h. A computer-controlled universal testing machine (Model 5942, Instron, Norwood, MA, USA) was used to fracture the specimens in three-point flexure. The other prepared specimens were subjected to thermocycling between 5 and 55 ℃ for 850 cycles with a dwell time of 45 s. After the thermocycling process, the flexural strength and modulus of the samples were evaluated using the same procedure at a span length of 20 mm and crosshead speed of 1 mm/min. Flexural strength (*σ*) and modulus (*E*) were calculated using the following equations:

1 $$\upsigma = \frac{3Fl}{2b{h}^{2}}$$

2 $$E= \frac{P{l}^{3}}{4b{h}^{3}d}$$

where *F* is the maximum load exerted on the specimen (N), *l* is the distance between the supports (20 mm), *b* and *h* are the width and height (mm) of the specimen, respectively, measured immediately before the test, *P* is the load at a point in the straight-line region of the load–displacement curve (N), and *d* is the deflection at load *P* (mm).

### Micro hardness

Five disc-shaped specimens (diameter: 10 mm; height: 2 mm) were prepared from each composite resin group using a metal mold. The sample from each group was placed in a Vickers hardness tester (MMT-X, Matsuzawa Seiki Co., Tokyo, Japan), and a 100 g load was applied for 15 s using a Vickers diamond indenter. The indentation was observed, and the Vickers hardness number (VHN) was measured. Measurements were made at three random sites for each specimen, and the mean values and standard deviations were calculated and compared. The remaining disc specimens were subjected to thermocycling between 5 and 55 ℃ for 850 cycles with a dwell time of 45 s. Then, the micro hardness of the thermocycled samples was measured using the same procedure.

### Color

Disc-shaped specimens with 10 mm diameter and 2.0 mm height were prepared (n = 5), and the color of the specimens was measured before and after thermocycling according to the CIE L*a*b* color scale relative to the standard illuminant, D65 using a spectrophotometer (CM-3500d; Konica Minolta, Sensing Inc., Osaka, Japan)^28^. In the CIELAB system, the specific shaded position of the color space is defined using three coordinates, viz., L*, a*, and b*. L* represents the lightness of color and can range between 0 (dark) and 100 (light), a* represents the color on a red (positive)–green (negative) axis, and b* represents the color on a yellow (positive)–blue (negative) axis. The measurements were repeated three times for each specimen. Then, all specimens were thermocycled between 5 and 55 ℃ for 850 cycles with 45 s dwelling time. Finally, the color of the thermocycles specimens was measured using the same procedure. Differences in the color parameters (ΔE00) were calculated for each experimental group using the CIEDE2000 color difference.

### Antibacterial properties

Disc-shaped specimens of the experimental and control group were prepared using a mold with a diameter of 10 mm and thickness of 2 mm. *S. mutans* (ATCC 25,175) was used as the bacterial specimen for evaluating the antibacterial activities of the composite resins. The bacterium was cultured in brain heart infusion (BHI) broth at 37 °C for 24 h. Then, its optical density (OD) at 600 nm (OD600) was adjusted to 0.05 and the diluted bacterial suspension was placed directly in the disc samples (n = 5). After 24 h of incubation, the disc samples were gently washed twice with phosphate-buffered saline to remove non-adherent bacteria. The adherent bacteria were harvested in 1 mL of BHI broth by sonication (SH − 2100; Saehan Ultrasonic, Seoul, Korea) for 5 min, and 100 μL of the suspension was seeded in a 96-well plate to measure the OD600 value using a microplate reader (Epoch, BioTek Instruments, VT, USA). The OD values relative to those of the untreated *S. mutans* suspension were also recorded. Moreover, the viability of the adherent bacteria was determined by staining using a live/dead microbial viability kit (Molecular Probes, Eugene, OR, USA), according to the manufacturer’s protocols. After 15 min of incubation at room temperature in the dark, the stained samples were observed by confocal laser microscopy (CLSM, LSM900; Carl Zeiss, Thornwood, NY, USA).

### Ion release from the resin composites

Five disc-shaped specimens (diameter: 10 mm; thickness: 2 mm) were prepared using a metal mold and then photoactivated by irradiating for 20 s with a curing unit. Subsequently, the specimens from each group were immersed in 5 mL of distilled water and stored for 7, 15, and 30 days at 37 ℃ in incubators. Then, the specimens were removed from water and the concentrations of the calcium, phosphorus, and strontium ion in the immersion solvent (water) were measured using inductively coupled plasma optical emission spectrometry (Optima 8300, PerkinElmer, Waltham, MA, USA). Other specimens were subjected to thermocycling between 5 and 55 ℃ with a dwell time of 45 s. The concentrations of the different ions released into deionized water from the thermocycled samples were also measured as described for as-cured samples.

### Cell viability

The cell viability was analyzed using a WST-1 assay for quantification of the cleavage of WST-1 (4-[3-(4-Iodophenyl)-2-(4-nitro-phenyl)-2H-5-tetrazolio]-1,3-benzene sulfonate) by mitochondrial dehydrogenases. Briefly, a total of 10,000 cells per well were seeded in a 96-well culture plate in 100 μL of cell culture medium and incubated at 37 ℃ under a 5% CO_2_ atmosphere. After 24 h of incubation, the medium was replaced by 100 μL eluate from each group. After further incubation in the humidified CO_2_ incubator at 37 °C for 24 h, 10 µL of WST-1 reagent was added, and the cells were incubated for additional two hours before reading the optical density (OD). The absorbance was measured at a wavelength of 450 nm using a microplate spectrophotometer (Epoch, BioTek, Winooski, VT, USA).

The cell viability was calculated as the percentage of the test group OD vs. the negative control OD. Cell viability was evaluated according to ISO 10,993–5.

### Statistical analysis

All statistical analyses were performed using statistical software IBS SPSS version 25.0 (IBM Korea Inc., Seoul, Korea); data from at least three independent experiments were used in the analysis. The results obtained for all groups were analyzed using one-way analysis of variance (ANOVA), followed by the Tukey's post-hoc test. In all analyses, the level of statistical significance was set at *p* < 0.05.

## Results

### Characterization of the Sr-PBG powder

**Figure 1:**
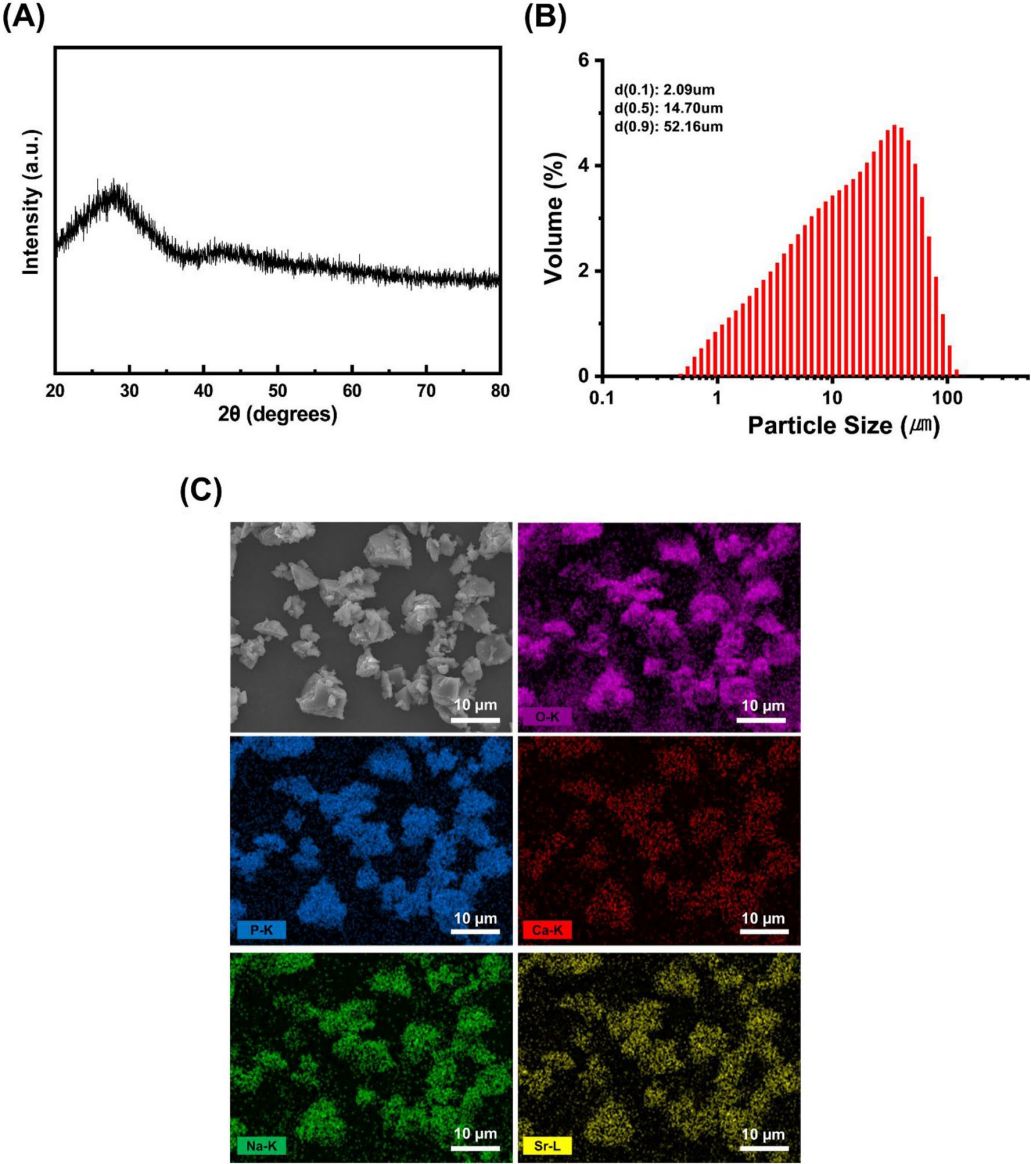
Characterization of the strontium-modified phosphate-based glass (Sr-PBG). (**A**) XRD pattern, (**B**) particle size distribution, and (**C**) FE-SEM image and the corresponding EDX mapping images of the Sr-PBG powder. Scale bar is 10 µm.

The XRD pattern of the Sr-PBG powder is shown in Fig. 1A, which reveals the absence of sharp peaks, indicating the amorphous nature of the sample, which is consistent with the non-crystalline nature of typical glasses. Figure 1B shows the particle size distribution of the Sr-PBG powder. Its particle size ranged from 2.09 to 52.16 μm with the median diameter (d50) being 14.70 μm. Figure 1C shows the morphologies of the Sr-PBG microparticles. The corresponding EDX mapping images reveal that phosphorus, calcium, sodium, strontium, and oxygen were homogeneously distributed throughout the sample.

### Flexural strength and modulus

**Figure 2:**
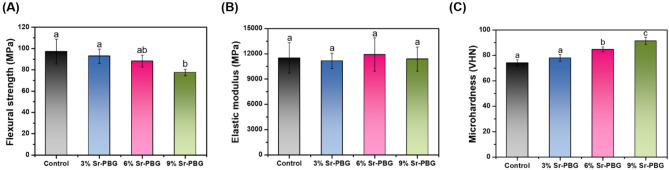
Mechanical properties of the strontium-modified phosphate-based glass (Sr-PBG) samples before thermocycling: (**A**) flexural strength, (**B**) elastic modulus, and (**C**) Vickers hardness. Different lowercase letters above the bars indicate significant differences, as determined by the Tukey's post-hoc test and the error bars represent the standard deviation of the mean.

**Figure 3:**
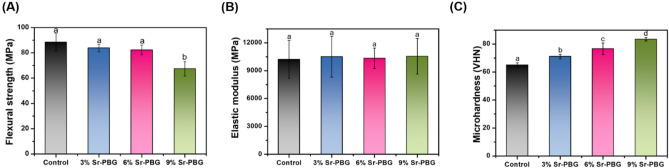
Mechanical properties of the strontium-modified phosphate-based glass (Sr-PBG) samples after thermocycling: (**A**) flexural strength, (**B**) elastic modulus, and (**C**) Vickers hardness. Different lowercase letters above the bars indicate significant differences, as determined by the Tukey's post-hoc test and the error bars represent the standard deviation of the mean.

Figures 2A,B compare the flexural strengths and elastic moduli, respectively, of the control and Sr-PBG groups measured before thermocycling, while Fig. 3A,B compare the same parameters measured after thermocycling. As shown in Fig. 2A, the flexural strengths of the 3% Sr-PBG and 6% Sr-PBG groups (92.81 ± 6.60, and 88.24 ± 5.66 MPa, respectively) were not significantly different from that of the control group (97.16 ± 11.70 MPa), whereas that of the 9% Sr-PBG group was significantly lower than that of the control group (p < 0.05). Further, as shown in Fig. 2B, the elastic moduli of the composite resins from the different groups did not differ significantly. The flexural strengths and elastic moduli of the thermocycled samples exhibited similar trends (Fig. 3A,B), and there were no significant differences between the elastic moduli of the control and Sr-PBG composite resins.

### Micro hardness

Figures 2C and 3C compare the micro hardness values of the control and experimental groups measured before and after thermocycling, respectively. The results reveal significant differences between the micro hardness values of the control group and experimental groups, with the exception of the 3% Sr-PBG group. The micro hardness of the control was 74.16 ± 2.40 VHN, while those of the 3% Sr-PBG, 6% Sr-PBG, and 9% Sr-PBG groups were 77.92 ± 2.62, 84.78 ± 1.92, 91.41 ± 2.78 VHN, respectively. Thus, the control group had the lowest micro hardness (p < 0.05). The addition of Sr-PBG affected the surface micro hardness of all the experimental groups. The same trend was maintained after thermocycling between the Sr-PBG groups (Fig. 3C).

### Color

The L* (lightness), a* (red-to-green axis), and b* (yellow-to-blue axis) values of the composite resins are summarized in Table 2. The control group had the lowest L* value. The L* values of the experimental groups were significantly higher than that of the control group (*p* < 0.05), and there were also significant differences between the L* values of the experimental groups (*p* < 0.05) before and after thermocycling. In addition, the control group exhibited significant differences in a* and b* values (*p* < 0.05) as compared with all other groups both before and after thermocycling. The magnitudes within the total CIEDE2000 color differences before and after thermocycling of the specimens are shown in Table 3.

**Table 2:** Color values of the control group and various experimental groups incorporating Sr-PBG before and after thermocycling.

Thermocycling	Groups	L*	a*	b*
Before	Control	55.81 ± 0.25^a^	− 0.30 ± 0.07^a^	3.19 ± 0.42^a^
	3% Sr-PBG	58.63 ± 0.37^b^	− 0.08 ± 0.09^b^	4.22 ± 0.27^b^
	6% Sr-PBG	59.35 ± 0.72^c^	− 0.03 ± 0.07^bc^	4.45 ± 0.33^b^
	9% Sr-PBG	60.93 ± 0.30^d^	0.04 ± 0.06^c^	4.78 ± 0.30^c^
After	Control	55.63 ± 0.33^a^	− 0.21 ± 0.78^a^	2.70 ± 0.36^a^
	3% Sr-PBG	59.14 ± 0.84^b^	0.04 ± 0.08^b^	3.51 ± 0.45^b^
	6% Sr-PBG	60.45 ± 0.89^c^	0.13 ± 0.69^c^	3.59 ± 0.51^b^
	9% Sr-PBG	61.90 ± 0.53^d^	0.13 ± 0.04^c^	3.91 ± 0.37^b^

The same lowercase letter in the same column indicates no significant difference (*p* > 0.05).

**Table 3:** Comparison of experimental composites before (A) and after (B) thermocycling with color differences (ΔE00).

Groups	ΔE (A)	ΔE (B)	ΔE (A-B)
Control	–	–	0.65 ± 0.35^a^
3% Sr-PBG	2.77 ± 0.48^a^	3.35 ± 0.49^a^	1.19 ± 0.46^a^
6% Sr-PBG	3.44 ± 1.04^a^	4.49 ± 1.09^ab^	1.46 ± 1.07^a^
9% Sr-PBG	4.83 ± 0.34^b^	5.75 ± 0.53^b^	1.22 ± 0.30^a^

The same lowercase letter in the same column indicates no significant difference (*p* > 0.05).

### Antibacterial properties

**Figure 4:**
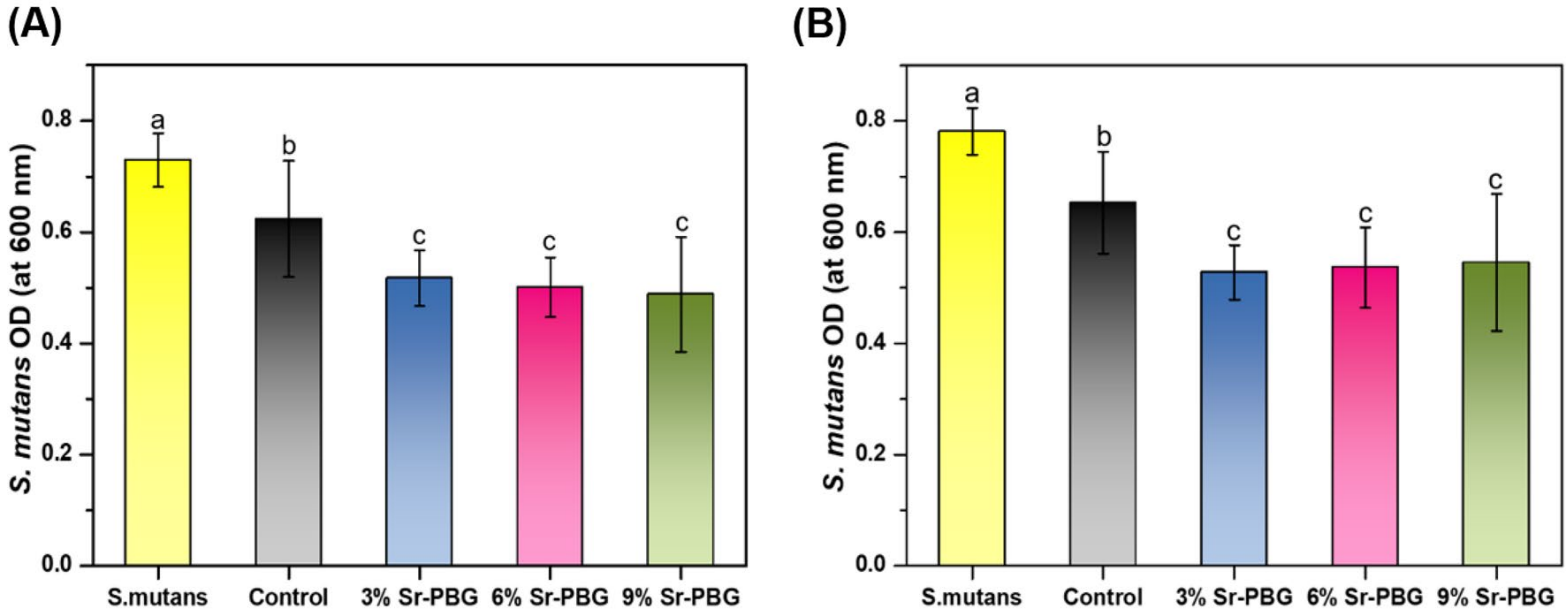
Comparison of the optical density (OD) values of *S. mutans* at 600 nm after incubation on the composite resins: (**A**) before thermocycling and (**B**) after thermocycling. Different lowercase letters above the bars indicate significant differences, as determined by the Tukey's post-hoc test and the error bars represent the standard deviation of the mean.

**Figure 5:**
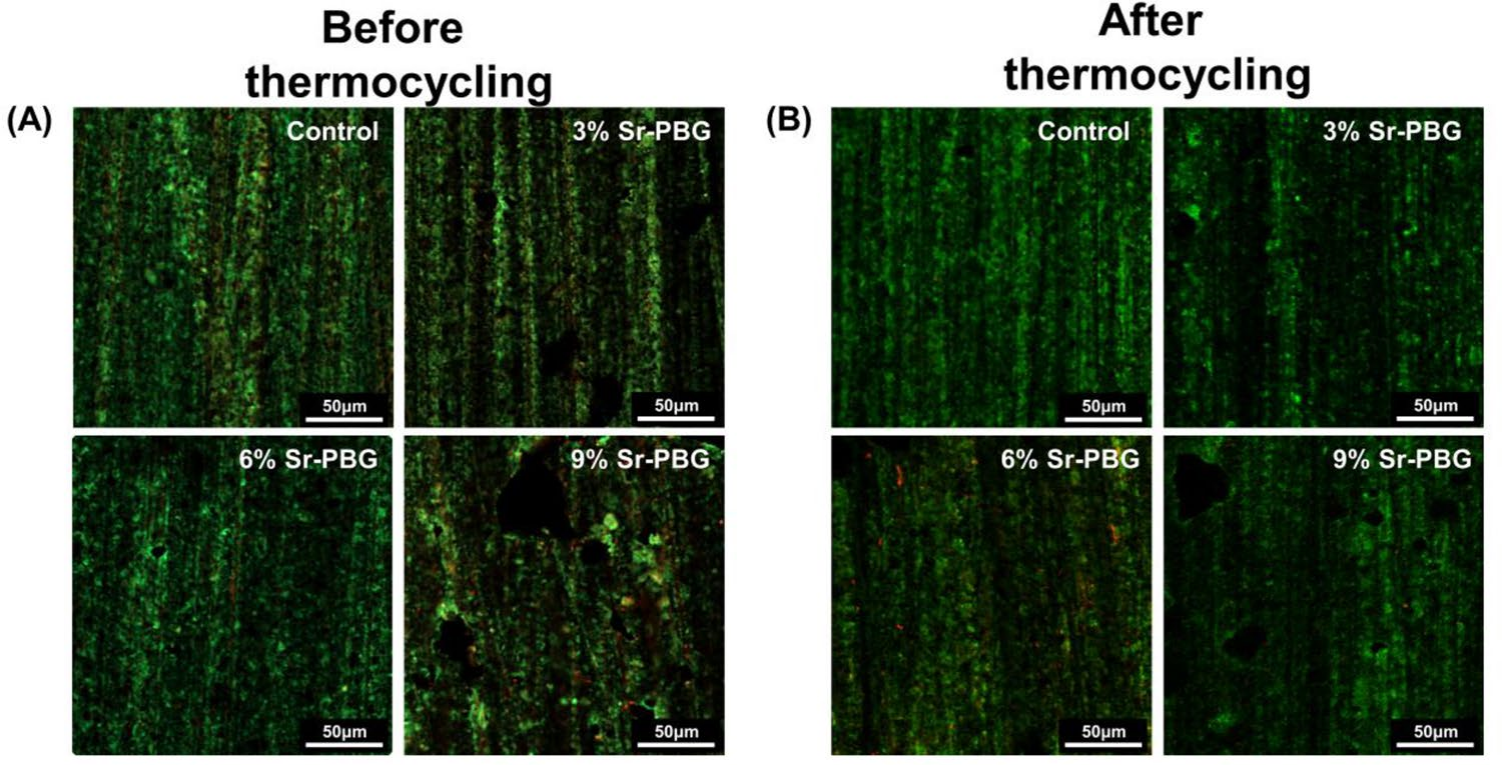
Live/dead staining images of bacteria attached on the surfaces (**A**) before and (**B**) after thermocycling.

Figure 4 presents the OD value of *S. mutans* measured after the incubation of the bacterial suspensions on different groups of composite resins (control and experimental group). The control group led to the highest OD (0.62 ± 0.10) of *S. mutans*, and the results from the control and experimental groups exhibited significant differences (*P* < 0.05). A similar trend was found following the thermocycling process, and there were significant differences between the results obtained with the control and experimental groups (Fig. 4B) (*P* < 0.05). The results of the live/dead assay showed the same trend for the OD value of *S. mutans* (Fig. 5).

### Ion release

**Figure 6:**
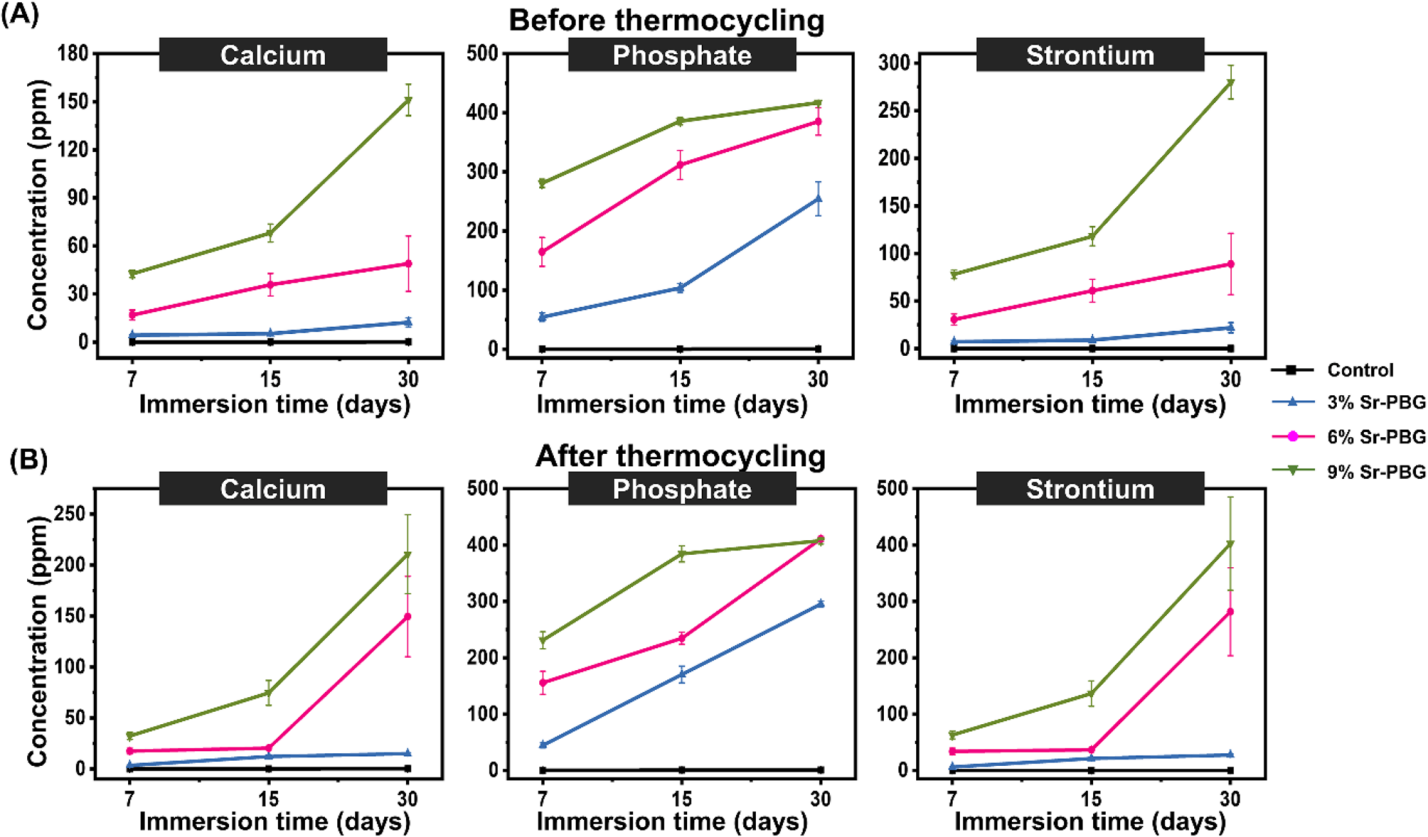
Ion release characteristics of the composite resins. Concentrations of the major ions (Ca, P, and Sr) released from each composite resin sample (**A**) before thermocycling and (**B**) after thermocycling. The error bars represent the standard deviation of the mean.

The amounts of Ca, P, and Sr ions eluted from the control and experimental groups of composite resins into deionized water over 7, 15, and 30 days were measured before and after thermocycling, and the results are shown in Fig. 5. As shown, the release of Ca and P from the composite resin increased as the amount of Sr-PBG in the sample increased (Fig. 6). There was no Sr release from the control group as it did not contain Sr. For all ions measured, significant differences were observed between the results obtained from the control group and 9% Sr-PBG group both before and after thermocycling (p < 0.05).

### Cell viability

**Figure 7:**
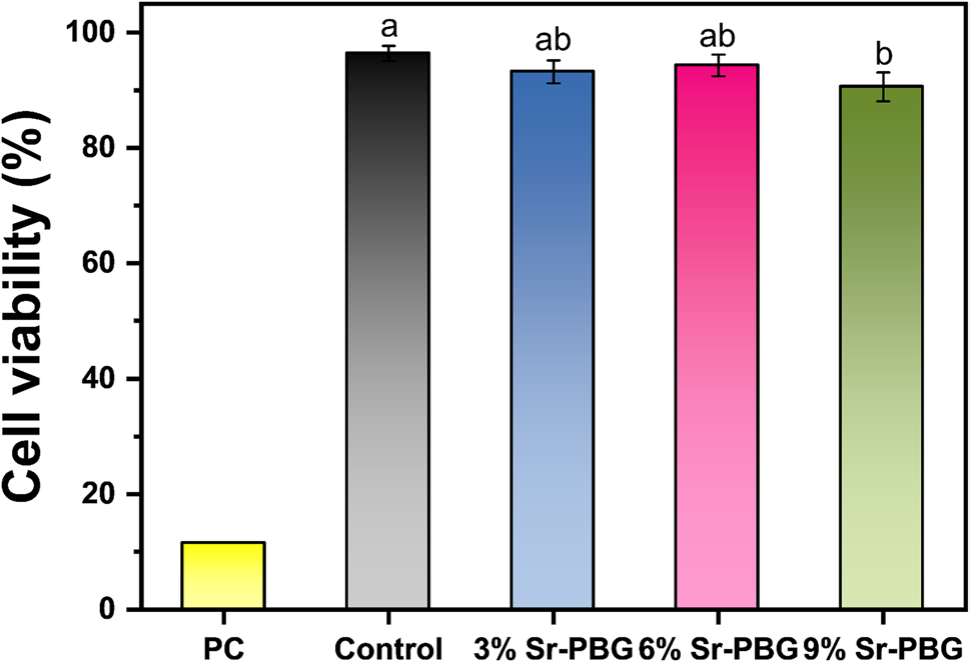
Cell viability of the composite resins. Different lowercase letters above the bars indicate significant differences, as determined by the Tukey's post-hoc test and the error bars represent the standard deviation of the mean.

The cell viability results obtained using the L-929 mouse fibroblasts are shown in Fig. 7. In the figure, the cell viability of the 3% Sr-PBG and 6% Sr-PBG groups are not significantly different from that of the control group. However, the 9% Sr-PBG group is significantly lower than that of the control group (p < 0.05). The cell viability is 96.41 ± 1.30 for the control group, 93.20 ± 1.97 for the 3% Sr-PBG group, 94.32 ± 1.87 for the 6% Sr-PBG group, and 90.59 ± 2.50 for the 9% Sr-PBG group.

## Discussion

Polymer resin/inorganic filler-based dental composite resins are widely used in dentistry because of their aesthetic superiority, high bonding strength as a cement, and mechanical reinforcement^29^. However, as the composite resin has little or no antibacterial activity, bacterial accumulation adjacent to the restoration margin can lead to secondary caries and ultimately shorten the lifespan of the composite restoration^24^. Recently, many studies have attempted to impart activity against *S. mutans* to composite resins by the incorporation of various antimicrobial additives^20, 24, 30^. However, the durability of the added antibacterial agent was questionable, and the mechanical properties of the composite resin with the added antibacterial agent were insufficient for long-term applications, and the antibacterial activity lasted only for a short time^31^. The production of composite resins with long-term antibacterial properties would be clinically beneficial. Therefore, this study considered the development of a novel composite resin that exhibits long-term antibacterial activity against *S. mutans* while maintaining the original favorable physical and mechanical properties. To determine the amount and concentration (wt%) of the SR-PBG filler, a preliminary test was conducted, and the concentration was set at 3, 6, and 9%. In terms of mechanical properties, at 9wt% or more, it was set within 9wt%, far outside the minimum allowable value of ISO 4049. When the concentrations of 1 and 2 wt% were added, there was no significant difference from the control group in terms of the antibacterial effect; thus, the starting concentration was 3%.

The evaluation of the mechanical properties (e.g., flexural strength, elastic modulus, and Vickers hardness) and long-term stability of oral conditions in vivo is essential to clarify the clinical potential of dental material development. The laboratory condition must mimic oral conditions to evaluate the clinical performance of dental materials. Therefore, thermocycling simulating abnormal changes in oral temperature was performed at 5 to 55 °C with a residence time of 45 s for 850 cycles corresponding to one month. As more than 9% of the Sr-PBG microfiller load was added before thermal cycling, the bending strength of the resin composite decreased. Aggregation is related to the dispersion of filler particles, which plays an important role in determining flexural properties^32^. Increased filler content leads to extensive filler agglomeration in the matrix. Therefore, it seems that the flexural strength was reduced due to the occurrence of propagating cracks because of the concentration of stress during loading. The modulus of elasticity indicates the resistance to the masticatory force applied to the oral restoration. The modulus of elasticity was not affected even when the filler content increased^33^. The micro hardness of the composites improved with increasing filler content, likely due to the addition of Sr-PBG as a microfiller because inorganic particles generally have a higher stiffness than the polymer matrix.

The mechanical properties of flexural strength, elastic modulus, and hardness of the composite resin decreased by 6 ~ 13%, 10 ~ 12%, and 8 ~ 12%, respectively, after thermal cycling aging. This is likely to be reduced by thermal cycle aging, resulting in water infiltration into the polymer resin matrix and hydrolysis within the polymer network or filler/polymer interface and weak bonding between the resin structure and the filler. However, a Sr-PBG addition of up to 6% can meet the requirement of 80 MPa, the minimum acceptable value (flexural strength) recommended by ISO 4049 for restorative resin composites. The color stability of composite restorations is important to meet the increasing esthetic demands. ΔE_00_ values less than 1.8 and greater than 0.8 are considered clinically acceptable for color changes detectable by the human eye^34^. The control group and the three experimental groups had ΔE_00_ values of 0.48 to 1.23 after thermocycling, which were all within the clinically acceptable range.

After incubation on the control group, the number of *S. mutans* was very high, as confirmed by the OD value. However, the OD value of *S. mutans* decreased with increasing Sr-PBG content in the composite resin. These results are similar to those obtained with silver-, zinc-, and gallium-doped bioactive glass additives^25, 37, 38^. Strontium is a major mineral that acts as an antimicrobial agent against *S. mutans*^20^. This effect is due to the influence of Sr cations, which increase the pH of the surrounding^39^. Furthermore, it has been suggested that the binding action of Ca, P, Na, and Si ions caused an anabolic reaction^40^.

The ion release results revealed that the concentrations of the Ca, P, and Sr ions released from the experimental groups were significantly higher than those from the control group. The results of this study reveal a correlation between the OD600 of *S. mutans* and the amount of Sr ions released from the composite resin; the count of *S. mutans* decreased as the concentration of the Sr ion increased.

In this study, Sr-PBG added to the composite resin could prevent secondary caries through two mechanisms. The first mechanism is based on an increase in osmotic pressure due to free release and ion dissolution, creating an environment in which bacteria cannot grow; this leads to an unfavorable increase in pH for the bacteria^41^. Additionally, released and dissolved Sr ions can cause enzyme modification such as blockage and loss of function, leading to bacterial death^42^. However, only one bacterial species, *S. mutans*, was used in this study. To further verify the antibacterial effect of the composite resin, long-term studies using various bacterial samples are needed. The second mechanism is based on the release of ions associated with remineralization. The Ca and P ions released from the PBG can enhance tooth remineralization and prevent mineral loss^40^.

The findings of this study provide insights that can aid the development of composite resins with long-term antibacterial effects in the future. However, in this study, in vitro tests were not performed using complex models in clinical oral environments, such as under the flow of saliva and in the presence of food residues. We plan to conduct cytotoxicity studies and in vitro tests in the future to evaluate their clinical use.

## Conclusion

In this study, composite resins added with a small percentage of Sr-PBG (3–9 wt.%) as a microfiller were fabricated, and their mechanical properties and long-term antibacterial activity against *S. mutans* were evaluated. The major conclusions drawn from this study are as follows:

1. The Sr-PBG microfiller can be successfully incorporated in the resin without deteriorating its flexural strength, modulus, and micro hardness.
2. The Sr-PBG-containing composite resin exhibits antibacterial activity and inhibits the growth of *S. mutans*.
3. The quantities of Ca, P, and Sr ions released increase as the Sr-PBG content in the composite resin increases.

The incorporation of Sr-PBG into the composite resin inhibits the growth of *S. mutans* without deteriorating the overall mechanical properties of the resin. Therefore, Sr-PBG is considered a potential filler for antibacterial dental restorative materials.

## Data Availability

The data will be shared at a reasonable request to the corresponding author.
